# A pilot investigation on DNA methylation modifications associated with complex posttraumatic symptoms in elderly traumatized in childhood

**DOI:** 10.1186/s13104-017-3082-y

**Published:** 2017-12-19

**Authors:** Zoya Marinova, Andreas Maercker, Edna Grünblatt, Tomasz K. Wojdacz, Susanne Walitza

**Affiliations:** 1 Department of Child and Adolescent Psychiatry and Psychotherapy, Psychiatric Hospital, University of Zurich, Zurich, Switzerland; 2 Department of Psychology, Division of Psychopathology and Clinical Intervention, University of Zurich, Binzmühlerstrasse 14/17, Raum BIN 3 E 14, 8050 Zurich, Switzerland; 3 Aarhus Institute of Advanced Studies, University of Aarhus, Aarhus, Denmark

**Keywords:** Childhood trauma, Complex posttraumatic stress disorder, DNA methylation, Epigenetic modifications

## Abstract

**Objective:**

Complex posttraumatic stress disorder (CPTSD) is a newly proposed diagnosis in the International Classification of Diseases-version 11, which is currently intensively investigated. Childhood trauma is regarded as main source of CPTSD symptoms, even in later life. Induction of DNA methylation changes by childhood trauma may contribute to its long-lasting adverse health consequences. The current study analyzed the correlation of genome-wide DNA methylation profiles with complex posttraumatic sequelae in buccal epithelial cells from 31 elderly former indentured child laborers (Verdingkinder) using the Infinium Illumina 450k Human DNA methylation chip.

**Results:**

DNA methylation modifications indicated experiment-wide significant associations with the following complex posttraumatic symptom domains: dissociation, tension reduction behavior and dysfunctional sexual behavior. Differentially methylated CpG sites were mapped to the genes huntington associated protein 1 (*HAP1*), RAN binding protein 2 (*RANBP2*) and proteasome subunit alpha 4 (*PSMA4*), respectively. In addition, the methylation of cg07225277 located in carnosine synthase 1 (*CARNS1*) correlated with trauma symptom complexity. Our pilot data suggest correlation of DNA methylation modifications with complex posttraumatic symptoms in elderly individuals subjected to prolonged and complex childhood trauma. More comprehensive and elaborated studies should be carried out to analyze epigenetic modifications associated with CPTSD.

## Introduction

Exposure to childhood adversities has been associated with increased risk for mental illness in later life, including posttraumatic stress disorder (PTSD) and complex posttraumatic stress disorder (CPTSD) [1, 2]. Symptoms characteristic of PTSD include re-experiencing the traumatic event, avoiding stimuli associated with it and hyperarousal. CPTSD according to the International Classification of Diseases-version 11 (ICD-11) incorporates in addition to the PTSD symptoms also disturbances in other mental and behavioral symptom areas [3, 4]. A high number of different childhood traumatic events has been correlated to increased complexity of posttraumatic symptoms in adulthood [5, 6].

DNA methylation is a mechanism of epigenetic regulation, which affects gene transcription. Various environmental factors including stress can alter the DNA methylation pattern of cells [7]. Childhood adversities have been associated with differential DNA methylation of genes involved in neuronal development, cell signaling, inflammation and disease biomarkers by several whole-genome studies [8]. Genome-wide DNA methylation studies in PTSD patients have implicated among others genes involved in immune function [9]. However, the link between DNA methylation changes and complex posttraumatic symptoms has not been specifically investigated.

Swiss former indentured child laborers (Verdingkinder) were removed as children from their families by the authorities due to different reasons (poverty, being born out of wedlock) and were placed to live and work on farms. This was a practice applied until the 1950s and many of the Verdingkinder were subjected to childhood trauma and neglect during the indentured labor [10]. We have previously identified differentially methylated genes in elderly former child laborers compared to a control group, which included genes involved in neuronal projections and neuronal development [11].

In the current investigation, we assessed the correlation of genome-wide DNA methylation profiles with complex posttraumatic symptoms according to ICD-11 CPTSD in a group of elderly Swiss former indentured child laborers.

## Main text

### Methods

#### Participants

31 elderly former indentured child laborers (17 M/14 F) were included in the current study. Mean age (± SD) was 76.4 (± 6.3) years. Mean age of indenture (± SD) was 4.7 (± 4.5) years. Mean duration of indenture (± SD) was 11.8 (± 5) years. The participants were part of a larger investigation analyzing mental and physical sequelae in elderly former indentured child laborers [12]. Ethical approval was granted by the Cantonal Ethic Commission of Zurich (KEK-ZH-Nr. 2012-0245). Inclusion criteria for the study were: indentured child labor, upbringing in rural areas of Switzerland, at least 65 years of age, voluntary participation, (Swiss) German speaking. Exclusion criteria were: under 65 years of age, insufficient knowledge of German.

#### Assessment of complex posttraumatic symptoms

Complex posttraumatic symptoms were evaluated with the trauma symptom inventory (TSI) [13–15]. TSI is a 100-item self-report instrument, which includes 10 clinical scales to assess multiple complex posttraumatic sequelae dimensions (anxious arousal, depression, anger and irritability, intrusive experiences, defensive avoidance, dissociation, sexual concerns, dysfunctional sexual behavior, impaired self reference and tension reduction behavior), as well as three validity scales and twelve critical items. Items are rated on scales ranging from 0 (never) to 3 (often) depending on how often symptoms occurred in the previous 6 months. The validation of the German version of the TSI was carried out in a group of former indentured child laborers and showed overall good reliability and validity [14].

To assess symptom complexity, TSI scores were dichotomized as previously described [5]. For each of the 10 clinical scales values at least 1.5 standard deviations above the median for all former indentured child laborers included in our project were considered as clinically elevated. The number of clinically elevated TSI scales for each participant were used as an indicator of symptom complexity [5].

#### DNA isolation, bisulfite conversion and Infinium Illumina 450k chip analysis

Buccal swabs collection and DNA stabilization was carried out as previously described [11]. Buccal swabs were processed at the “Barts and The London Genome Centre” in the United Kingdom. DNA isolation was performed with the Isohelix buccal DNA isolation kit (Cell Projects Ltd) and purification with the Zymo ZR-96 DNA clean-up kit (Zymo Research Corporation, Irvine, United States) according to the manufacturer’s recommendations. DNA concentration was controlled with the Qubit 2.0 Fluorometer (Life Technologies) and integrity with agarose gel electrophoresis. DNA bisulfite conversion was carried out with the EZ DNA Methylation kit (Zymo Research Corporation) according to the manufacturer’s recommendations. Bisulfite converted DNA was hybridized to the Infinium Illumina 450k Human DNA methylation chip, which encompasses 485,000 methylation sites per sample [16].

#### Array analysis

Analysis was carried out in the R environment (http://cran.r-project.org) using raw intensity data files [17]. Data import, quality control and normalization were carried out with the *minfi* Bioconductor package [18]. Probes with detection p-values > 0.01 in any of the samples were excluded. Background correction and normalization were carried out with the Illumina method implemented in *minfi* (bg.correct = TRUE, normalize = “controls”). Probes on the Illumina Infinium 450k array assaying single nucleotide polymorphisms (SNPs) as well as non-CpG methylation were filtered out. So were the ones containing SNPs at the investigated CpG site or at a single base extension position. Probes located on the sex chromosomes were also excluded. The *minfi* preprocessed data were analyzed with the *CpGassoc* package to assess correlation of DNA methylation profiles with complex posttraumatic symptoms measured by the TSI scales [19]. Age, gender, the estimated proportion of buccal epithelial cells, array and slide were included as covariates in the analysis. The proportion of buccal epithelial cells was estimated according to a previously published protocol [20]. The threshold for statistical significance of the results was set at 5 × 10^−7^ according to a previously recommended threshold to account for multiple testing [21].

### Results

DNA methylation profiles of elderly former indentured child laborers were significantly associated (p < 5 × 10^−7^) with complex posttraumatic sequelae in the symptom dimensions dissociation, tension reduction behavior and dysfunctional sexual behavior (Table 1). No significant (p < 5 × 10^−7^) association of DNA methylation patterns were observed for the scales anxious arousal, depression, anger and irritability, intrusive experiences, defensive avoidance, sexual concerns and impaired self reference.

**Table 1:** Differentially methylated CpG sites, which reached experiment-wide significance

TSI Scale	CpG site	Chromosome	Gene symbol	p-value
Dissociation	cg12320221	17	*HAP1*	4.54 × 10^−7^
Tension reduction behavor	cg12328023	2	*RANBP2*	3.61 × 10^−7^
Dysfunctional sexual behavior	cg20739864	15	*PSMA4*	2.8 × 10^−7^
Number of clinically elevated scales	cg07225277	11	*CARNS1*	1.02 × 10^−7^

Methylation changes at cg12320221 mapped to the first exon of the gene huntington associated protein 1 (*HAP1*) correlated significantly and negatively with dissociation symptoms severity (*T*-statistic = − 11.45; *p* value = 4.54 × 10^−7^). Positive correlation of DNA methylation with the scale tension reduction behavior was detected for cg12328023, which is located in the body of the gene RAN binding protein 2 (*RANBP2; T*-statistic = 11.73; *p*-value = 3.61 × 10^−7^). For the scale dysfunctional sexual behavior positive correlation of DNA methylation was observed for cg20739864 in the 3′-UTR of the gene proteasome subunit alpha 4 (*PSMA4; T*-statistic = 12.05; *p*-value = 2.8 × 10^−7^).

Finally, association of DNA methylation with the number of clinically elevated TSI scales as an indicator of trauma symptom complexity was investigated. Positive correlation for DNA methylation at cg07225277 located in the body of carnosine synthase 1 (*CARNS1*) was found (*T*-statistic = 13.41; *p*-value = 1.02 × 10^−7^).

### Discussion

Our pilot investigation as part of a comprehensive psychobiologial research program [11, 12] identified DNA methylation alterations associated with the complex posttraumatic symptom domains dissociation, tension reduction behavior and dysfunctional sexual behavior in elderly former indentured child laborers. Experiment-wide significant association of DNA methylation modifications with trauma symptom complexity was also observed.

For the dissociation scale differential methylation in *HAP1* was identified. *HAP1* has been shown to be critically involved in a number of neuronal functions, including neuronal survival, inhibitory synaptic transmission and neurogenesis [22, 23]. Disruption of *HAP1* in the early postnatal period in mice has been associated with depressive-like behavior in later life [24].

*RANBP2* (with DNA methylation correlated with tension reduction behavior) is involved in SUMOylation, a posttranslational modification with important role in a range of neuronal functions, including neuronal survival and synaptic transmission [25]. *PSMA4*, methylation in which was associated with dysfunctional sexual behavior, is also involved in posttranslational modifications, since it encodes a subunit of the 20S proteasome. The ubiquitin–proteasome system plays an important role in the regulation of memory formation [26].

Correlation of DNA methylation in carnosine synthase 1 (*CARNS1*, known also as *ATPGD1*) with trauma complexity was also observed. *CARNS1* catalyzes the formation of carnosine and homocarnosine, which are compounds with anti-oxidant properties [27]. Dietary supplementation with carnosine has shown positive effects on cognition on veterans suffering from Gulf War illness [28].

Overall, we detected stronger association of DNA methylation profiles with complex posttraumatic symptoms rather than “classical” PTSD symptoms. DNA methylation alterations were found in genes involved in neuronal function and posttranslational modifications. Proving the relevance of these findings would require their replication in larger study cohorts and after different trauma types.

Our pilot findings imply a correlation of DNA methylation modifications with complex posttraumatic sequelae in elderly individuals exposed to prolonged and complex childhood trauma.

## Limitations

Limitations of our investigation include its cross-sectional design, the relatively small sample size, the use of peripheral tissue (buccal epithelial cells), the lack of data on gene expression patterns for the genes showing alterations in DNA methylation and the use of a CPTSD assessment that is not fully equivalent to the ICD-11 CPTSD model [see 29]. Although certain tissue specificity in epigenetic patterns exists, evidence that DNA methylation alterations after early life adversity is a system-wide phenomenon has been accumulating, supporting the utility of surrogate peripheral tissues for analysis [30].

## Abbreviations

PTSD: posttraumatic stress disorder
CPTSD: complex posttraumatic stress disorder
HAP1: huntington associated protein 1
RANBP2: RAN binding protein 2
PSMA4: proteasome subunit alpha 4
CARNS1: carnosine synthase 1
ICD-11: International Classification of Diseases-version 11
M: male
F: female
SD: standard deviation
TSI: trauma symptom inventory
SNP: single nucleotide polymorphism
